# Proper paraffin slide storage is crucial for translational research projects involving immunohistochemistry stains

**DOI:** 10.1186/2001-1326-3-4

**Published:** 2014-03-17

**Authors:** Mary Economou, Liliane Schöni, Caroline Hammer, José A Galván, Dominique-Elisabeth Mueller, Inti Zlobec

**Affiliations:** 1 Translational Research Unit (TRU) Institute of Pathology University of Bern, Murtenstrasse 31, Room L313, CH-3010 Bern, Switzerland

**Keywords:** Translational research, Paraffin slide, Tissue microarray, Storage, Immunohistochemistry

## Abstract

The use of paraffin slides and tissue microarrays (TMA) is indispensable for translational research. However, storage of paraffin slides over time has a substantial detrimental effect on the quality and reliability of immunohistochemistry stains. Particularly affected by this issue may be any collaborative efforts where paraffin slides or TMAs are shipped to central laboratories and then ‘biobanked’ for some time until use. This article summarizes some of the key issues affecting loss of antigenicity on paraffin slides and some simple storage solutions to help maintain high quality immunohistochemistry results when paraffin slides must be stored for a certain time prior to use.

## Background

Translational research is in large part based on the use of formalin-fixed paraffin embedded (FFPE) tissue blocks. When sectioned, tissue slides can be used for a variety of purposes such as special stains, immunohistochemistry, and in situ hybridization to study morphology, protein expression, DNA aberrations and RNA expression. Tissue microarrays (TMAs) can also be constructed from FFPE material. These are produced by the repeated transfer of small tissue cores from a series of tissue blocks into a single recipient ‘TMA’ block [1]. TMA slides have the advantage of concentrating a large number of tissues on a minimal number of slides, reducing costs, resources, and tissue usage [2].

Although histological slides are invaluable for research, it is important to underline that paraffin slides lose their value with longer storage times, particularly for immunohistochemistry applications. The timing of tissue block sectioning followed by their use is paramount in maintaining the quality and reliability of results.

### Longer storage time is detrimental to antigenicity

Longer storage times can considerably affect the quality of immunohistochemistry staining, resulting in false-negative findings [3]. The longer the storage time at room temperature (RT) is, the worse the effects on antigenicity [3-5]. Storage time affects loss of nuclear, cytoplasmic and membranous biomarkers and impacts whole tissue sections and TMAs alike [4,6]. Already at 3 months at RT, Ki-67 and other biomarkers show substantial losses in the percentage of positive cells and staining intensities compared to freshly cut slides [3-5], underlining the importance of planning and timing research studies using this type of material.

### Other reasons for loss of antigenicity

In addition to time, several other reasons affect the quality of immunohistochemistry on paraffin slides in storage. Exposure of the slides to the air leads to oxidation which impacts immunoreactivity [7]. Hydrolysis, namely exposure of tissues to endogenous water resulting from inadequate tissue processing has a major impact on antigen degradation [8]. Exposure to sun or light [9] diminishes antigenicity as does storage of paraffin slides at higher temperatures [10,11]. Storage of slides at 4°C is shown in several studies to help prevent antigen degradation [5,10-12] but may also be sub-optimal due to the humid environment and thus hydrolysis occurring from exogenous water [8]. Although not related to storage per se, it should be mentioned that inappropriate and prolonged fixation may damage antigenicity or cause diffusion artefacts, and that these effects may differ from one antigen to another [13]. Of course, the type of fixative also has an important impact on antigenicity [14]. Summarizing, the following factors may have a considerable impact on the quality of immunohistochemistry:

– Storage time

– Oxidation

– Hydrolysis

– Tissue processing time

– Sun or light exposure

– Fixation time

– Type of fixative

### Paraffin slide storage solutions

**Figure 1:**
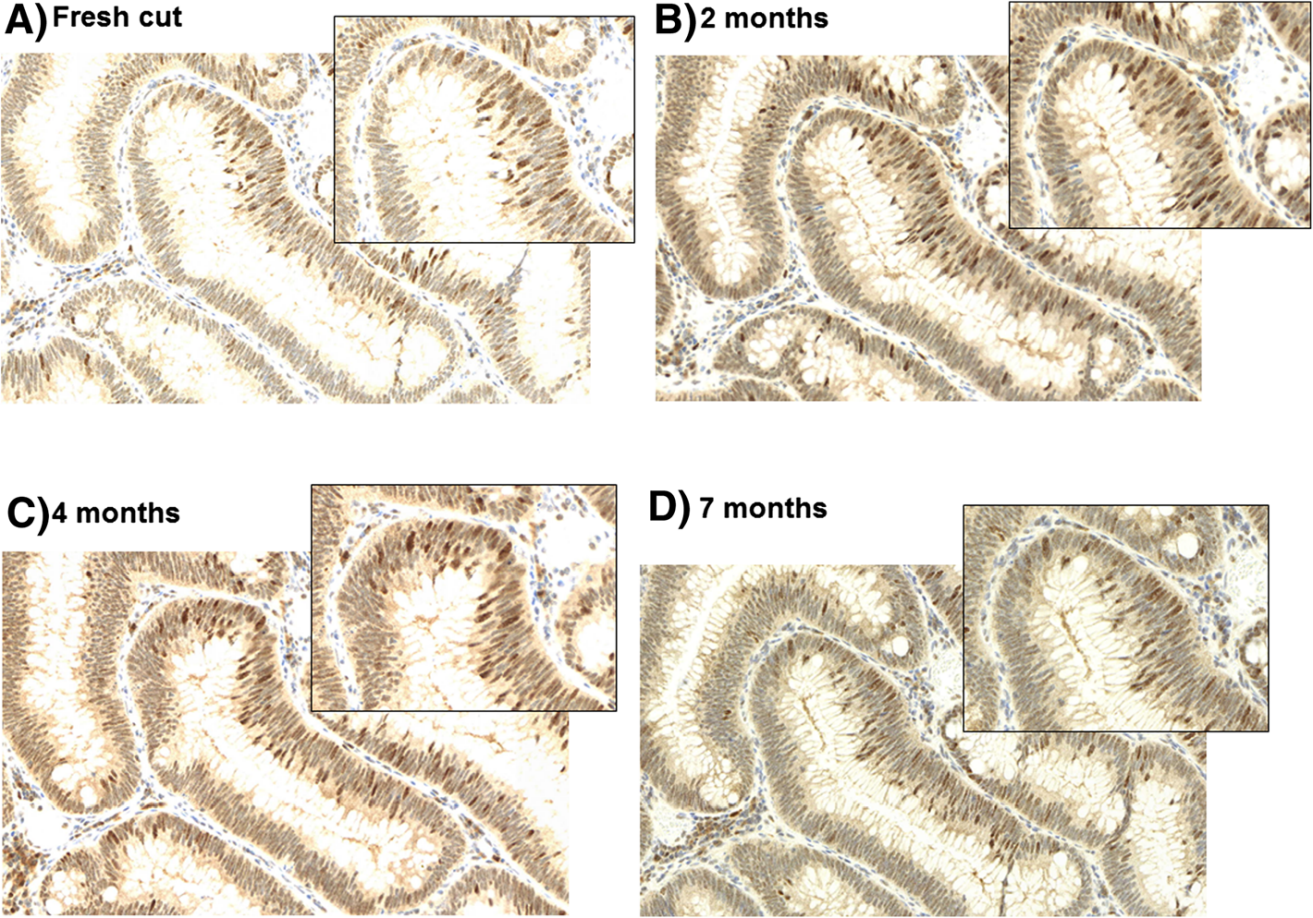
Immunohistochemistry staining of a colorectal adenoma with mismatch repair protein MSH6 (20×; annotated region 40×) A) on a freshly cut slide (0 months), after paraffin-coated storage B) after 2 months, C) after 4 months and D) after 7 months.

In our laboratory, dipping freshly cut slides after 1 day of drying into paraffin is a routine practice for both whole tissue sections and TMAs. Coating or dipping with paraffin ‘seals’ the tissue and should reduce oxidation. Slides are ready to use after melting to remove the additional wax in an incubator. Standard immunostaining protocols are followed thereafter. In combination with a cool environment, e.g. at 4°C, paraffin-coated slides should provide adequate short-term storage of tissues. Several studies support this solution. Gelb and colleagues evaluated PTEN staining in 26 breast cancers by investigating storage at RT on uncoated slides, RT with coated slides, 4° uncoated slides and 4°C with coated slides and show that the latter is ideal for antigen preservation after 4 months [12]. DiVito and colleagues have also shown the superiority of coated paraffin slides for antigen preservation but recommend additional nitrogen storage [4]. The combination of uncoated slides at ambient temperatures is harmful to antigenicity, particularly with longer storage time [15,16]. DiVito underlines that slides under such conditions stained for Ki-67 produce unreadable results in some instances. Our own evidence suggests that slide coating is effective for whole tissue sections and TMAs in most, but not all, cases for at least several months and may depend on protein abundance. In Figure 1, tissue slides from a colorectal adenoma were preserved by slide dipping followed by immunohistochemistry for MSH6 at 2 months, 4 months and 7 months. Staining shows little variation in comparison to a slide stained soon after sectioning.

## Discussion

The issue of slide storage and detrimental effect on antigenicity may be of particular relevance in some settings. In any context where tissue slides are ‘biobanked’ for a certain time prior to use, for example in international collaborative studies, the effect of storage time must be considered. The problem may be magnified if slides from local institutions are shipped to central laboratories for immunohistochemistry. Translational projects stemming from clinical trials may involve FFPE material. These tissues are requested from pathology institutes whose policy may dictate that tissue slides rather than paraffin blocks are forwarded. Slides may be shipped off to another laboratory for further testing. A delay between sectioning and immunohistochemistry may lead to false-negative results. In the case of large cohort studies, investigators may like to have slides from each case sectioned prior to use, however this could lead to qualitative differences in staining between sections cut earlier or later. Ambitious research projects that have not been adequately organized may suffer the consequences of poor quality or unreadable stains if slides are not used relatively quickly after sectioning.

## Conclusion

The quality of immunohistochemistry results on paraffin slides is affected by storage time and several other factors. It is imperative that studies be adequately planned so that fresh sections of paraffin blocks can be used as much, and as soon as possible. If sections are coated, it may be important to perform standardized quality control assessment for collaborative studies. Results from immunohistochemistry stains may be maintained for several months by using some simple techniques such as coating/dipping freshly cut slides into paraffin and storing these at 4°C, but may be marker-dependent. Storage of cut slides at RT over time without additional precautions should be avoided.

## Competing interests

The authors declared that they have no competing interests.

## Authors’ contributions

ME: conceived and designed the study and performed histological experiments. LS: provided expert histological support. CH, JG, and DEM: provided technical support and performed immunohistochemistry. IZ: designed the study and drafted the manuscript. All authors reviewed, edited and approved the final version of the manuscript.

## References

[B1] Kononen J Bubendorf L Kallioniemi A Barlund M Schraml P Leighton S Torhorst J Mihatsch MJ Sauter G Kallioniemi OP Tissue microarrays for high-throughput molecular profiling of tumor specimens Nat Med 1998 3 844 847 10.1038/nm0798-844 9662379

[B2] Zlobec I Koelzer VH Dawson H Perren A Lugli A Next-generation tissue microarray (ngTMA) increases the quality of biomarker studies: an example using CD3, CD8, and CD45RO in the tumor microenvironment of six different solid tumor types J Transl Med 2013 3 104 10.1186/1479-5876-11-104 23627766 PMC3644251

[B3] Bertheau P Cazals-Hatem D Meignin V de Roquancourt A Verola O Lesourd A Sene C Brocheriou C Janin A Variability of immunohistochemical reactivity on stored paraffin slides J Clin Pathol 1998 3 370 374 10.1136/jcp.51.5.370 9708203 PMC500697

[B4] DiVito KA Charette LA Rimm DL Camp RL Long-term preservation of antigenicity on tissue microarrays Lab Invest 2004 3 1071 1078 10.1038/labinvest.3700131 15195116

[B5] Wester K Wahlund E Sundstrom C Ranefall P Bengtsson E Russell PJ Ow KT Malmstrom PU Busch C Paraffin section storage and immunohistochemistry. Effects of time, temperature, fixation, and retrieval protocol with emphasis on p53 protein and MIB1 antigen Appl Immunohistochem Mol Morphol 2000 3 61 70 10.1097/00022744-200003000-00010 10937051

[B6] Fergenbaum JH Garcia-Closas M Hewitt SM Lissowska J Sakoda LC Sherman ME Loss of antigenicity in stored sections of breast cancer tissue microarrays Cancer Epidemiol Biomarkers Prev 2004 3 667 672 15066936

[B7] Blind C Koepenik A Pacyna-Gengelbach M Fernahl G Deutschmann N Dietel M Krenn V Petersen I Antigenicity testing by immunohistochemistry after tissue oxidation J Clin Pathol 2008 3 79 83 17412873 10.1136/jcp.2007.047340

[B8] Xie R Chung JY Ylaya K Williams RL Guerrero N Nakatsuka N Badie C Hewitt SM Factors influencing the degradation of archival formalin-fixed paraffin-embedded tissue sections J Histochem Cytochem 2011 3 356 365 10.1369/0022155411398488 21411807 PMC3201147

[B9] Ramos-Vara JA Webster JD Dusold D Miller MA Immunohistochemical evaluation of the effects of paraffin section storage on biomarker stability Vet Pathol 2014 3 102 109 10.1177/0300985813476067 23435571

[B10] Jacobs TW Prioleau JE Stillman IE Schnitt SJ Loss of tumor marker-immunostaining intensity on stored paraffin slides of breast cancer J Natl Cancer Inst 1996 3 1054 1059 10.1093/jnci/88.15.1054 8683636

[B11] van den Broek LJ van de Vijver MJ Assessment of problems in diagnostic and research immunohistochemistry associated with epitope instability in stored paraffin sections Appl Immunohistochem Mol Morphol 2000 3 316 321 10.1097/00022744-200012000-00009 11127924

[B12] Gelb AB Freeman VA Astrow SH Evaluation of methods for preserving PTEN antigenicity in stored paraffin sections Appl Immunohistochem Mol Morphol 2011 3 569 573 10.1097/PAI.0b013e318217a3d3 21552118

[B13] Khoury T Sait S Hwang H Chandrasekhar R Wilding G Tan D Kulkarni S Delay to formalin fixation effect on breast biomarkers Mod Pathol 2009 3 1457 1467 10.1038/modpathol.2009.117 19734848

[B14] Nietner T Jarutat T Mertens A Systematic comparison of tissue fixation with alternative fixatives to conventional tissue fixation with buffered formalin in a xenograft-based model Virchows Arch 2012 3 259 269 10.1007/s00428-012-1248-5 22814649 PMC3432218

[B15] Prioleau J Schnitt SJ p53 antigen loss in stored paraffin slides N Engl J Med 1995 3 1521 1522 10.1056/NEJM199506013322217 7739705

[B16] Vis AN Kranse R Nigg AL van der Kwast TH Quantitative analysis of the decay of immunoreactivity in stored prostate needle biopsy sections Am J Clin Pathol 2000 3 369 373 10.1309/CQWY-E3F6-9KDN-YV36 10705817

